# Risk of retinal artery occlusion in patients with diabetes mellitus: A retrospective large-scale cohort study

**DOI:** 10.1371/journal.pone.0201627

**Published:** 2018-08-09

**Authors:** Yuh-Shin Chang, Chung-Han Ho, Chin-Chen Chu, Jhi-Joung Wang, Sung-Huei Tseng, Ren-Long Jan

**Affiliations:** 1 Department of Ophthalmology, Chi Mei Medical Center, Tainan, Taiwan; 2 Graduate Institute of Medical Science, College of Health Science, Chang Jung Christian University, Tainan, Taiwan; 3 Department of Medical Research, Chi Mei Medical Center, Tainan, Taiwan; 4 Department of Hospital and Health Care Administration, Chia Nan University of Pharmacy and Science, Tainan, Taiwan; 5 Department of Anesthesiology, Chi-Mei Medical Center, Tainan, Taiwan; 6 Department of Recreation and Health-Care Management, Chia-Nan University of Pharmacy and Science, Tainan, Taiwan; 7 Department of Ophthalmology, National Cheng Kung University Hospital, College of Medicine, National Cheng Kung University, Tainan, Taiwan; 8 Department of Pediatrics, Chi Mei Medical Center, Liouying, Tainan, Taiwan; Universidad Francisco de Vitoria, SPAIN

## Abstract

There is a globally increasing prevalence and incidence of diabetes mellitus (DM). Prolonged hyperglycaemia could lead to both macrovascular damage, such as carotid artery atherosclerosis, and microvascular damage, such as retinal arteriolar narrowing, and might contribute to retinal artery occlusion (RAO). Accordingly, it is important to determine whether DM is a contrubuting factor of RAO. We conducted a retrospective cohort study that included 241,196 DM patients from the Longitudinal Cohort of Diabetes Patients Database who were recruited between 2003 and 2005. An age- and sex-matched non-DM control group included the same number of patients who were selected from the Taiwan Longitudinal Health Insurance Database of 2000. Relevant data of each patient were collected from the index date until December 2013. The incidence and risk of RAO were calculated and compared between the DM and non-DM groups. The hazard ratio for RAO was calculated using Cox proportional hazards regression analysis after adjusting for confounders. The cumulative incidence rate of RAO was calculated by Kaplan–Meier analysis. In total, 317 patients with DM and 144 controls developed RAO during the follow-up period, leading to an incidence rate of RAO in DM patients that was 2.30 times (95% confidence interval [CI] = 1.89–2.80) greater than that in controls. After adjustment for potential confounders, patients with DM were 2.11 times (95% CI, 1.71–2.59) more likely to develop RAO in the total study cohort. In conclusion, DM increases the risk of RAO, which is an interdisciplinary emergency. Close collaboration between endocrinologists and ophthalmologists is important in managing RAO following DM.

## Introduction

The increasing prevalence of diabetes mellitus (DM), a group of physiological dysfunctions characterized by chronically blood glucose elevation related to insulin resistance and inadequate insulin or glucagon secretion, is an important public health problem worldwide[1–3]. The public health burden of DM is because DM and its accompanying complications due to sustained hyperglycaemia are rapidly increasing, resulting in morbidity and mortality worldwide. These complications are classified as macrovascular complications, including stroke and ischaemic heart disease, and microvascular complications, including retinopathy, neuropathy, and nephropathy [4–6]. The eye is one of the principal organs affected by DM, with ocular complications related to the disease a leading cause of blindness and becoming a general public health issue [7–9]. While diabetic retinopathy is the most common and well-known microvascular ocular complication, it is not the only ocular retinal complication [10, 11]. Retinal vessel occlusion is another ocular retinal disorder that is seen in patients with DM because of the common pathogenic mechanisms.

Retinal artery occlusion (RAO), including various types of central RAO (CRAO) and branch RAO (BRAO), is one of the leading causes of profound and permanent visual impairment [12, 13]. Despite the low incidence rate of RAO, CRAO (incidence rate = 1.64 per 100,000 person-years), and BRAO (incidence rate = 4.99 per 100,000 person-years) [14], RAO is an important emergent ocular problem due to the acute severe loss of vision and subsequently increased risk of stroke or acute coronary syndrome [14, 15]. RAO, analogous to stroke in the brain, is caused by acute occlusion of the retinal artery, which is a branch of the ophthalmic artery originating from the intracranial branch of the internal carotid artery [12, 13]. The most common pathophysiology of RAO is embolism, which is usually one of three types (cholesterol, calcific, or platelet-fibrin), that commonly arises from thrombi or ulcerated atherosclerotic plaques within the carotid arteries or less commonly from cardiac valvular structures [12, 13]. Additionally, platelet aggregation on carotid atherosclerotic plaques may release serotonin, which may contribute to occlusion of retinal blood flow and play a role in RAO development [12, 16]. Furthermore, haemodynamically related retinal ischemia, due to a dramatic reduction in ocular blood flow, may occur as a result of internal carotid artery stenosis or narrowed retinal arterioles [12, 13, 16].

Several reports have shown that DM is an independent risk factor for atherothrombotic ischaemic heart disease and stroke [17–19] and that the two major pathological processes occurring in the arterial wall of patients with DM are arterial stiffening and atherosclerotic change, which could lead to atherothrombotic disorders [20–22]. Atherosclerotic plaques in the carotid artery are linked to both embolism and serotonin release, which play important roles in the development of RAO. Song et al. showed that the total plaque area and mean carotid intima–media thickness are significantly greater in patients with RAO than in the general population [23]. In addition, retinal microvascular abnormalities with defective retinal microcirculation, such as focal or generalized arteriolar narrowing and arteriovenous nicking, are prominent pathological features and frequently reported in patients with DM [24, 25]. Meanwhile, several studies have shown that focal or generalized arteriolar narrowing and arteriovenous nicking are common microvascular retinopathic findings in patients with RAO [13, 26, 27]. In addition to their common pathogenic mechanisms, DM and RAO share some systemic risk factors, including hypertension, hyperlipidaemia, congestive heart failure, coronary artery disease, and chronic renal disease. Therefore, it is clinically relevant to determine whether DM is a predictor of RAO. However, no previous studies have investigated this association between DM and RAO. Therefore, we used a nationwide population-based dataset to design a cohort study for evaluating the association between DM and RAO in Taiwan.

## Methods

### Database

Taiwan launched a single-payer National Health Insurance (NHI) scheme, which has provided extensive medical care coverage for all residents in Taiwan since March 1, 1995. As of 2007, the program enrolled >98% of the total Taiwanese population of 22.96 million (i.e. 22.60 million individuals). The data for our cohort study were obtained from the Taiwan National Health Insurance Research Database (NHIRD), which supplies information regarding patient sex, date of birth, admission and discharge dates, and enciphered patient identification numbers. It also includes the International Classification of Diseases, Ninth Revision, Clinical Modification (ICD-9-CM), diagnoses and procedure codes, prescriptions details, and costs covered and paid by NHI. This study was granted exemption from review by the Institutional Review Board of Chi-Mei Medical Center. The requirement of informed consent was waived because analysing datasets from a database is devoid of identifiable personal information.

### Selection of patients and variables

Two study groups were enrolled in the retrospective cohort study, both recruited during 2003–2005: a new-onset DM group and a matched non-DM (control) group. In total, 241,196 patients with DM with the ICD-9-CM code 250 diagnosis made between January 1, 2003 and December 31, 2005 were included. They were selected from the Longitudinal Cohort of Diabetes Patients database (LHDB), which is a data subset of the NHIRD and consists of a random sample of 120,000 newly diagnosed patients per year from 1996 to 2013 with their complete medical records. Patients with unknown sex, missing data, or age <20 years were excluded, as were patients diagnosed with RAO [ICD-9-CM codes 362. 31 (CRAO) and 362.32 (BRAO)] before DM.

For each patient with DM, one control without DM was randomly selected from the longitudinal Health Insurance Database, 2000 (LHID2000), which is a data subset of the NHIRD that includes the overall claim data for one million beneficiaries (4.34% of the total population) randomly selected in 2000. There was no significant difference in age, sex, or healthcare costs between the sample group and all NHI enrolees. The 241,196 controls were matched with the patients with DM by sex, age, and index date. The index date for the patients with DM was the date of their initial diagnosis, while that for the controls was matched with the DM patient’s index date. Controls diagnosed with DM or RAO before the index date were excluded. Each patient was followed up to determine the incidence of RAO until the end of 2013 or death, whichever came earlier.

To distinguish patients who developed RAO after DM, every patient was tracked from his or her index outpatient visit or hospitalization until December 2013 and their demographic data were recorded (e.g. age and sex). Furthermore, we collected data for comorbidities, including hypertension (ICD-9-CM codes 401–405), hyperlipidaemia (ICD-9-CM code 272), congestive heart failure (ICD-9-CM code 428), coronary artery disease (ICD-9-CM code 410–414), and chronic renal diseases (ICD-9-CM code 582–588 except 587 and 584), because these conditions are critical risk factors for RAO [12]. In this study, the inclusion criterion for hypertension, hyperlipidaemia, congestive heart failure, coronary artery disease, and chronic renal disease was as follows: documentation of the condition at least once in the inpatient setting or ≥3 times in the ambulatory setting within 1 year before the index date.

### Statistical analysis

SAS 9.4 for Windows (SAS Institute, Inc., Cary, NC, USA) was used for statistical analyses. Pearson’s chi-square tests were used to compare the demographic characteristics and comorbidities between the DM and control groups. The incidence of RAO was calculated as the number of RAO patients identified during the follow-up period divided by the total number of person-years (PY) for each group by age, sex, and selected comorbidities. The incidence rate ratio (IRR), which represented a comparison of the RAO risk between the DM and control groups, was calculated by Poisson regression analysis. Kaplan-Meier analyses were performed to calculate the cumulative incidence rates for RAO, and log-rank tests were used to analyse the differences in the cumulative incidence rate curves. Cox proportional hazards regression was used to calculate the adjusted hazard ratios (HRs) for developing RAO. The data are presented as means [standard deviations (SDs)], and 95% confidence intervals (CIs) are provided where applicable. Kaplan-Meier curves were generated using STATA (version 12; Stata Corp. College Station, TX). Statistical significance was defined as P < 0.05.

## Results

### Demographic data

Between 2003 and 2005, 241,196 DM patients and 241,196 controls were recruited after excluding ineligible subjects. Table 1 provides the demographic data for the patients with DM and the age- and sex-matched controls. Data for the evaluated comorbidities are also presented in Table 1. The mean age of the DM and control patients was 55.06 (SD, 14.86) years. Of the 241,196 DM patients, 134,213 (55.64%) were men and 106,983 (44.36%) were women, with 91,921 (38.11%) aged 20–50 years, 86,120 (35.71%) 50–64 years, and 63,155 (26.18%) ≥65 years. Regarding the comorbidities, including hypertension, hyperlipidaemia, congestive heart failure, coronary artery disease, and chronic renal disease, the DM group exhibited a significantly higher prevalence compared with the control group.

**Table 1:** Demographic characteristics and co-morbid disorders in the diabetes mellitus and control groups.

	Diabetes Mellitus (N = 241,196)	Control (N = 241,196)	
	n (%)	n (%)	P-value
Age (years), mean ± SD	55.06 ± 14.86	55.06 ± 14.86	1.0000
Age (years)			
20–50	91,921 (38.11)	91,921 (38.11)	1.0000
50–64	86,120 (35.71)	86,120 (35.71)	
≥65	63,155 (26.18)	63,155 (26.18)	
Gender			
Male	134,213 (55.64)	134,213 (55.64)	
Female	106,983 (44.36)	106,983 (44.36)	1.0000
Baseline comorbidities			
Hypertension	74,645 (30.95)	26,280 (10.90)	<0.0001
Hyperlipidaemia	25,108 (10.41)	6,026 (2.50)	<0.0001
Congestive heart failure	6,521 (2.70)	1,846 (0.77)	<0.0001
Coronary heart disease	21,612 (8.96)	7,709 (3.20)	<0.0001
Chronic renal disease	6,021 (2.50)	2,213 (0.92)	<0.0001

Note: The demographic characteristics and comorbid disorders in the diabetes mellitus and control groups were compared using Pearson chi-square tests.

### Incidence rates for RAO

During the follow-up period, 461 (461/482,392, 0.095%) patients developed RAO, with the proportion being significantly higher in the DM group (317/241,196, 0.13%) than in the control group (144/241,196, 0.059%; Table 2). In addition, there was a significant difference in the RAO incidence rate (DM, 1.49/10000 PY; control, 0.65/10000 PY) and IRR (2.30, 95% CI = 1.89–2.80, P < 0.0001; Table 2) between the two groups.

**Table 2:** Risk of retinal artery occlusion (RAO) in the diabetes mellitus and control groups.

	Diabetes Mellitus				Control				Incidence Rate Ratio (95% CI)	P-value
	N	RAO	Person-years	Rate*	N	RAO	Peason-years	Rate*		
All	241,196	317	2,123,243.1	1.49	241,196	144	2,218,269.0	0.65	2.30 (1.89–2.80)	<0.0001
Central retinal artery occlusion		188 (59.31)	2,123,853.1	0.89		86 (59.72)	2,218,475.7	0.39	2.28 (1.77–2.95)	<0.0001
Branch retinal artery occlusion		129 (40.69)	2,124,114.9	0.61		58 (40.28)	2,218,675.3	0.26	2.32 (1.70–3.17)	<0.0001
Age (years)										
20–50	91,921	65	850,509.0	0.76	91,921	16	866,779.6	0.18	4.14 (2.40–7.15)	<0.0001
50–64	86,120	130	777,200.9	1.67	86,120	53	795,910.3	0.67	2.51 (1.83–3.46)	<0.0001
≥65	63,155	122	495,533.2	2.46	63155	75	555,579.1	1.35	1.82 (1.37–2.43)	<0.0001
Sex										
Male	134,213	211	1,163,244.6	1.81	134,213	89	1,227,415.2	0.73	2.50 (1.95–3.20)	<0.0001
Female	106,983	106	959,998.5	1.10	106,983	55	990,853.8	0.56	1.99 (1.44–2.76)	<0.0001
Baseline comorbidities										
Hypertension	74,645	121	632,136.4	1.91	26,280	44	228,286.7	1.93	0.99 (0.70–1.40)	0.9687
Hyperlipidaemia	25,108	43	223,928.2	1.92	6,026	12	53,959.4	2.22	0.86 (0.46–1.64)	0.6530
Congestive heart failure	6,521	13	46,976.9	2.77	1,846	0	14,503.2	-	-	-
Coronary heart disease	21,612	51	177,116.9	2.88	7,709	10	66,115.8	1.51	1.90 (0.97–3.75)	0.0627
Chronic renal disease	6,021	14	42,292.4	3.31	2,213	3	16,920.9	1.77	1.87 (0.54–6.50)	0.3264

Note: A Poisson regression analysis was performed to calculate the incidence rate ratio.

*Rate: per 10,000 person-years.

Next, we classified RAO into CRAO and BRAO. The majority of RAO cases in both groups were CRAO, including 188 of 317 (59.31%) and 86 of 144 (59.72%) patients with CRAO in the DM and control groups, respectively. There was a significant difference in the CRAO incidence (DM, 0.89/10000 PY; control, 0.39/10000 PY) and IRR (2.28; 95% CI = 1.77–2.95; P < 0.0001; Table 2) between the two groups. Regarding BRAO, there were 129 patients (40.69%) in the DM group and 58 (40.28%) in the control group (2.32; 95% CI = 1.70–3.17; P < 0.0001; Table 2).

Concerning the three age groups, patients with DM aged ≥65 years exhibited the highest incidence of RAO (2.46/10000 PY), followed by those aged 50–64 years (1.67/1000 PY) and 20–50 years (0.76/1000 PY). The IRR values were significantly higher for the three DM age groups than for controls within the same age ranges (Table 2). In particular, the incidence was 4.14 times higher in patients with DM aged 20–50 years than in controls of the same age (IRR = 4.14; 95% CI = 2.40–7.15; P < 0.0001).

The incidence rate of RAO was 1.81/10000 PY for the male patients with DM and 0.73/10000 PY for the male controls (IRR = 2.50; 95% CI = 1.95–3.20; P < 0.0001). A significant difference was also observed between the female patients with DM and controls (IRR = 1.99; 95% CI = 1.44–2.76; P < 0.0001; Table 2).

In the DM group, the incidence rates of RAO, from the highest to the lowest, were in the order of patients with chronic renal disease (3.31/10000 PY), coronary artery disease (2.88/10000 PY), congestive heart failure (2.77/10000 PY), hyperlipidaemia (1.92/10000 PY), and hypertension (1.91/10000 PY). The IRR for RAO in patients with DM with these comorbidities did not indicate a significantly greater risk compared with their controls.

**Fig 1:**
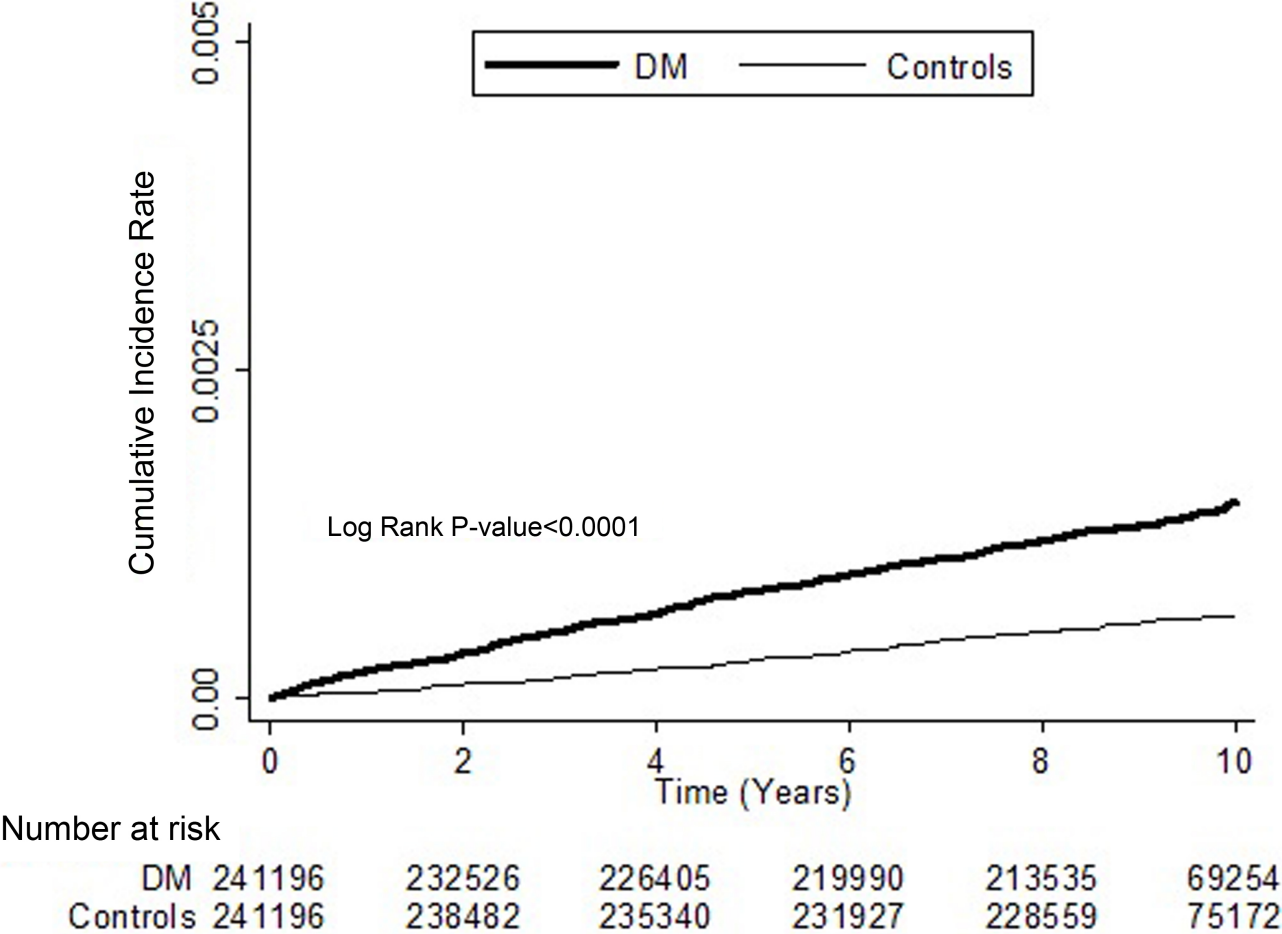
Cumulative incidence of retinal artery occlusion (RAO) in patients with diabetes mellitus (DM) and controls during the follow-up period.

Table 3 provides the crude and adjusted HRs for RAO during the follow-up period. After adjusting for age, sex, and the selected comorbidities, DM remained an independent risk factor for RAO (adjusted HR = 2.11; 95% CI = 1.71–2.59). Significant risk factors for RAO in both groups included an age of 50–64 years (adjusted HR = 2.40; 95% CI = 1.84–3.12; P < 0.05), age ≥65 years (adjusted HR = 3.59; 95% CI, 2.74–4.71; P < 0.05), male gender (adjusted HR, 1.59; 95% CI = 1.31–1.93; P < 0.05), and hypertension (adjusted HR, 1.24; 95% CI = 1.00–1.55; P < 0.05). Hyperlipidaemia, congestive heart failure, coronary artery disease, and chronic renal disease were not independent risk factors for RAO. Kaplan–Meier analyses revealed higher cumulative incidence rates of RAO in the DM group than in the control group; the findings of the log-rank tests were also significant (P < 0.0001; Fig 1).

**Table 3:** Crude and adjusted hazard ratios and 95% confidence interval (CI) calculated using the Cox proportional hazard regression for retinal artery occlusion during the follow-up period for the study cohort.

	Crude hazard ratio (95% CI)	Adjusted hazard ratio (95% CI)
Diabetes mellitus		
Yes	2.30 (1.89–2.80)*	2.11 (1.71–2.59)*
No	1.00	1.00.
Age (years)		
20–50	1.00	1.00
50–64	2.47 (1.90–3.20)*	2.40 (1.84–3.12)*
≥65	3.96 (3.06–5.13)*	3.59 (2.74–4.71)*
Sex		
Male	1.52 (1.25–1.84)*	1.59 (1.31–1.93)*
Female	1.00	1.00
Baseline comorbidities		
Hypertension		
Yes	2.25 (1.86–2.73)*	1.24 (1.00–1.55)*
No	1.00	1.00
Hyperlipidaemia		
Yes	1.98 (1.50–2.63)*	1.26 (0.94–1.70)
No	1.00	1.00
Congestive heart failure		
Yes	2.01 (1.16–3.49)*	0.89 (0.50–1.57)
No	1.00	1.00
Coronary heart disease		
Yes	2.56 (1.96–3.36)*	1.34 (0.99–1.79)
No	1.00	1.00
Chronic renal disease		
Yes	2.75 (1.69–4.46)*	1.52 (0.93–2.50)
No	1.00	1.00

Note: The adjusted hazard ratio for developing retinal artery occlusion was calculated using the Cox proportional hazard regression analysis.

*p-value <0.05.

## Discussion

Following a thorough review of the relevant literature, we found that no large-scale population-based study has been conducted to explore the relationship between DM and subsequent RAO. We analysed 241,196 patients with DM and 241,196 age- and sex-matched control subjects to examine this association. The study results indicated a significantly increased risk of RAO in patients with DM compared with controls and that DM was still an independent risk factor for RAO in the total sample after accounting for age, sex, and comorbidities including hypertension, hyperlipidaemia, congestive heart failure, coronary artery disease, and chronic renal disease.

To our knowledge, no study has previously shown an association between RAO and DM or investigated the pathophysiological association between these two conditions. Several common pathogenic mechanisms of RAO and DM, including macrovascular change, such as arterial stiffening and atherosclerosis change, and microvascular change, such as arterial stiffening and atherosclerotic change, will be discussed separately below.

The most well-known pathogenic mechanism common to both DM and RAO is macrovascular change, including arterial stiffening and atherosclerotic change. Many studies have demonstrated that DM strongly predisposes individuals to atherothrombotic cardiovascular disease [5, 17, 19]. The two major pathological changes in the arterial wall, arterial stiffening and susceptibility to atherosclerosis, may provide a functional and structural background for macrovascular disease. The aetiologies of arterial wall change in patients with DM are multifactorial and include many important factors. These include chronic hyperglycaemia, which can provoke structural and functional changes in the vascular wall by various mechanisms such as deterioration of antioxidant defence mechanisms [28, 29], overexpression of advanced glycation end-products, and enhancement of collagen cross-linking [30]; and insulin resistance, which is linked to increased oxidative stress [31–34], impairment of endothelial function impairment [31–33], and induction of low-grade inflammation [31, 32]. The presence of vascular biomarkers such as endothelial dysfunction, increased carotid intima-media thickness, plaque formation, and arterial stiffness, which are all associated with subclinical structural or functional impairment of the vascular wall [35]; and other DM-associated metabolic and systemic dysfunctions such as hypertension, hyperlipidaemia, and metabolic syndromes, which are linked to atherosclerosis, arterial stiffening, or both are also included. Meanwhile, the total plaque area and the mean carotid intima–media thickness were found to be higher not only in patients with DM but also in patients with RAO [17, 23]. Once arterial stiffening and susceptibility to atherosclerosis develops, emboli may form thrombi or ulcerated atherosclerotic plaques in the atherosclerosed carotid artery and contribute to the development of RAO because the retinal artery originates from the ophthalmic artery, which is the first intracranial branch of the internal carotid artery [12]. In addition, serotonin, which is released after platelet aggregation on atherosclerotic plaques in the carotid artery, may induce retinal artery vasospasm and retinal blood flow occlusion and participate in the pathophysiology of RAO [12, 16].

Another pathogenic mechanism common to both RAO and DM is microvascular retinopathy including narrower retinal arterioles. The retina, which requires copious oxygen to function, receives oxygen-rich blood from the ophthalmic artery and divides into a microvascular meshwork of arterioles. Persistent blood sugar elevation leads to biochemical changes over the microvascular meshwork resulting in basement membrane thickening and increased collagen deposition in the arterioles [36]. Several studies have suggested that microvascular retinopathy, including narrower retinal arterioles and arteriovenous nicking, is reflected by microvascular changes associated with not only hypertension, but also diabetes [25, 37, 38]. There have been several studies in patients with DM that have shown the association between narrower retinal arterioles and a higher risk of the incidence of diabetic retinopathy, stroke, and stroke-related mortality [24, 25, 39, 40]. Yang et al. showed that the presence of narrower retinal arterioles is significantly associated with increased intima-media thickness of the common carotid artery [41]. Furthermore, microvascular retinopathy is a common manifestation in both patients with DM and RAO. Although microvascular retinopathy is not the leading cause of RAO, microvascular retinopathy, including narrower retinal arterioles and arteriovenous nicking, may reflect generalized microvascular disease caused by vascular endothelial dysfunction [13, 26, 27], increase the susceptibility of the retinal vessels to occlusion, haemodynamically reduce ocular blood flow, and participate in RAO development.

The eldest group, aged ≥65 years, exhibited the highest incidence of RAO in both the DM and control groups (Table 2), and old age remained an important independent risk factor after adjusting for other confounding factors in the both groups (Table 3). Because atherosclerosis change in the macrovascular and microvascular environment is a chronic and continuous process, the age-dependent incidence rate trend was plausible and that the oldest population group had the highest incidence rate would be logical when considering the development of RAO.

We found that male sex is a significant risk factor in developing RAO after accounting for age and comorbidities in both groups (Table 3). This finding may be due to typical lifestyle differences between men and women. A higher proportion of men could live with high-risk lifestyle factors, such as smoking or alcohol consumption, which could not be adjusted in the NHIRD dataset and are well-known risk factors for atherosclerosis, the leading cause of RAO.

Some studies have reported that several comorbidities, such as hypertension, hyperlipidaemia, congestive heart failure, coronary artery disease, and chronic renal failure, are associated with RAO, a vision-threatening retinal vascular disease [12, 42–44]. In this cohort study, we evaluated these comorbidities in patients with DM and found that hypertension was the only significant risk factor for RAO in both groups. This finding is agreement with those in several previous reports showing hypertension to be a major risk factor for RAO [12, 44, 45]. Several studies disclosed that hypertension is linked to atherosclerosis, arteriosclerosis, retinal vessel wall damage, and thromboembolism, all of which result in the development of RAO [12, 44, 45]. Therefore, patients with DM and hypertension should be suitably advised about blood pressure control to ameliorate the risk of developing RAO.

RAO in DM is an interdisciplinary emergency and close collaboration between endocrinologists and ophthalmologists is necessary and important. Endocrinologists should be aware of the severe visual impairment that can be caused by RAO, which typically presents as sudden and painless monocular visual loss in patients with DM. The most important concerns for the ophthalmologist are distinguishing RAO from other causes of occlusive retinal vessel disease such as central retinal vein occlusion or branch retinal vein occlusion, and other causes of sudden, painless loss of vision such as ischaemic optic neuropathy, diabetic macular oedema, or vitreous haemorrhage. Although multiple therapies have been attempted, there is no well-established treatment guideline for RAO [46]. The multiple potential therapies for RAO include the use of ocular massage, which can manually dislodge emboli [47], carbogen or sublingual isosorbide dinitrate, which can dilate retinal vessels. The also include hyperbaric oxygen or hyperventilation, which could increase blood oxygen content [48]; intravenous acetazolamide or mannitol, topical intraocular pressure lowering agents or anterior paracentesis, which could increase the perfusion pressure gradient by reducing intraocular pressure [49]; and systemic aspirin or pentoxifylline, intravenous platelet inhibitor, or intra-arterial recombinant tissue plasminogen activator, which could contribute to thrombolysis [50]. When managing RAO in patients with DM, close cooperation between endocrinologists and ophthalmologists is important and reduces the risk of visual impairment.

Our study has several strengths. First, the nationwide, population-based study including a large sample of DM patients increases the statistical power and elevates the precision of the risk appraisal. Second, the selection bias in referral centres and chances of misdiagnosis are low because patients with visual disturbances visit ophthalmologists rather than general practitioners in Taiwan. Finally, our results are reliable because our study is a cohort study with longitudinal data obtained over the course of 10 years regarding RAO incidence and hypertension, hyperlipidaemia, congested heart failure, coronary artery disease, and chronic renal disease were considered as confounding factors when adjusting the HR for RAO in patients with DM.

This study also has some limitations. We cannot confirm if the controls had a history of ESRD before January 1996, because the sampled patients’ medical histories can only be traced back to the year 1996. In addition, several important confounding factors, including alcohol consumption, smoking history, and body mass index, could not be assessed. Furthermore, we could not obtain the current blood pressure and serum cholesterol levels, which may have introduced some bias. To decrease the effects of this problem, we included hypertension and hyperlipidaemia as confounding factors. Additionally, the diagnosis of DM, RAO, and other comorbidities relied on ICD-9-codes, which may have led to misclassification. Finally, the insurance claims data did not include information on the current blood sugar value or haemoglobin A_1C_ level, therefore we could not evaluate whether control of blood sugar levels influences the risk of developing RAO.

In summary, our study showed that the risk of RAO was significantly higher in patients with DM than in those without, and DM remained an independent risk factor after adjusting for hypertension, hyperlipidaemia, congestive heart failure, coronary artery disease, and chronic renal disease in the cohort. The association between DM and RAO is based on macrovascular changes, such as carotid artery atherosclerosis, which is associated with embolism formation, and microvascular changes, such as retinal arteriolar narrowing, which make the retinal vessels vulnerable to occlusion and contribute to RAO formation [13, 26, 27]. These results suggest that clinicians should educate patients with DM about RAO in addition to ensuring appropriate control of blood sugar levels. Close cooperation between endocrinologists and ophthalmologists is necessary to manage RAO following DM and prevent further visual impairment.

## References

[1] Blair M (2016) Diabetes Mellitus Review. Urologic nursing 36: 27–36 27093761

[2] Nathan DM (2015) Diabetes: Advances in Diagnosis and Treatment. Jama 314: 1052–1062 10.1001/jama.2015.9536 26348754

[3] Shaw JE, Sicree RA, Zimmet PZ (2010) Global estimates of the prevalence of diabetes for 2010 and 2030. Diabetes research and clinical practice 87: 4–14 10.1016/j.diabres.2009.10.007 19896746

[4] Shi Y, Vanhoutte PM (2017) Macro- and Microvascular Endothelial Dysfunction in Diabetes. Journal of diabetes 9: 434–449 10.1111/1753-0407.12521 28044409

[5] Orchard TJ, Costacou T (2016) Cardiovascular complications of type 1 diabetes: update on the renal link. Acta diabetologica 9: 325–334 10.1007/s00592-016-0949-7 27995339

[6] Khalil H (2017) Diabetes microvascular complications-A clinical update. Diabetes & metabolic syndrome 11 suppl 1: S133–S139 27993541 10.1016/j.dsx.2016.12.022

[7] Vieira-Potter VJ, Karamichos D, Lee DJ (2016) Ocular Complications of Diabetes and Therapeutic Approaches. BioMed research international 2016: 3801570 10.1155/2016/3801570 27119078 PMC4826913

[8] Sayin N, Kara N, Pekel G (2015) Ocular complications of diabetes mellitus. World journal of diabetes 6: 92–108 10.4239/wjd.v6.i1.92 25685281 PMC4317321

[9] Pinazo-Duran MD, Zanon-Moreno V, Garcia-Medina JJ, Arevalo JF, Gallego-Pinazo R, Nucci C (2016) Eclectic Ocular Comorbidities and Systemic Diseases with Eye Involvement: A Review. BioMed research international 2016: 6215745 10.1155/2016/6215745 27051666 PMC4808667

[10] Jeganathan VS, Wang JJ, Wong TY (2008) Ocular associations of diabetes other than diabetic retinopathy. Diabetes care 31: 1905–1912 10.2337/dc08-0342 18753669 PMC2518369

[11] Khan A, Petropoulos IN, Ponirakis G, Malik RA (2017) Visual complications in diabetes mellitus: beyond retinopathy. Diabetic medicine: a journal of the British Diabetic Association 34: 478–484 27917530 10.1111/dme.13296

[12] Hayreh SS, Podhajsky PA, Zimmerman MB (2009) Retinal artery occlusion: associated systemic and ophthalmic abnormalities. Ophthalmology 116: 1928–1936 10.1016/j.ophtha.2009.03.006 19577305 PMC2757505

[13] Hayreh SS (2011) Acute retinal arterial occlusive disorders. Progress in retinal and eye research 30: 359–394 10.1016/j.preteyeres.2011.05.001 21620994 PMC3137709

[14] Chang YS, Jan RL, Weng SF, Wang JJ, Chio CC, Wei FT, et al (2012) Retinal artery occlusion and the 3-year risk of stroke in Taiwan: a nationwide population-based study. American journal of ophthalmology 154: 645–652 e641 10.1016/j.ajo.2012.03.046 22809785

[15] Chang YS, Chu CC, Weng SF, Chang C, Wang JJ, Jan RL (2015) The risk of acute coronary syndrome after retinal artery occlusion: a population-based cohort study. The British journal of ophthalmology 99: 227–231 10.1136/bjophthalmol-2014-305451 25147366

[16] Hayreh SS, Piegors DJ, Heistad DD (1997) Serotonin-induced constriction of ocular arteries in atherosclerotic monkeys. Implications for ischemic disorders of the retina and optic nerve head. Arch Ophthalmol 115: 220–228 9046257 10.1001/archopht.1997.01100150222012

[17] Kozakova M, Palombo C (2016) Diabetes Mellitus, Arterial Wall, and Cardiovascular Risk Assessment. International journal of environmental research and public health 13: 201 10.3390/ijerph13020201 26861377 PMC4772221

[18] Beckman JA, Creager MA, Libby P (2002) Diabetes and atherosclerosis: epidemiology, pathophysiology, and management. Jama 287: 2570–2581 12020339 10.1001/jama.287.19.2570

[19] Booth GL, Kapral MK, Fung K, Tu JV (2006) Relation between age and cardiovascular disease in men and women with diabetes compared with non-diabetic people: a population-based retrospective cohort study. Lancet 368: 29–36 10.1016/S0140-6736(06)68967-8 16815377

[20] Colwell JA, Lopes-Virella M, Halushka PV (1981) Pathogenesis of atherosclerosis in diabetes mellitus. Diabetes care 4: 121–133 7009108 10.2337/diacare.4.1.121

[21] Martens FM, van der Graaf Y, Dijk JM, Olijhoek JK, Visseren FL (2008) Carotid arterial stiffness is marginally higher in the metabolic syndrome and markedly higher in type 2 diabetes mellitus in patients with manifestations of arterial disease. Atherosclerosis 197: 646–653 10.1016/j.atherosclerosis.2007.02.019 17374372

[22] Stehouwer CD, Henry RM, Ferreira I (2008) Arterial stiffness in diabetes and the metabolic syndrome: a pathway to cardiovascular disease. Diabetologia 51: 527–539 10.1007/s00125-007-0918-3 18239908

[23] Song YJ, Cho KI, Kim SM, Jang HD, Park JM, Kim SS, et al (2013) The predictive value of retinal vascular findings for carotid artery atherosclerosis: are further recommendations with regard to carotid atherosclerosis screening needed? Heart and vessels 28: 369–376 10.1007/s00380-012-0258-1 22684417

[24] Klein R, Klein BE, Moss SE, Wong TY, Hubbard L, Cruickshanks KJ, et al (2004) The relation of retinal vessel caliber to the incidence and progression of diabetic retinopathy: XIX: the Wisconsin Epidemiologic Study of Diabetic Retinopathy. Arch Ophthalmol 122: 76–83 10.1001/archopht.122.1.76 14718299

[25] Klein R, Klein BE, Moss SE, Wong TY (2007) Retinal vessel caliber and microvascular and macrovascular disease in type 2 diabetes: XXI: the Wisconsin Epidemiologic Study of Diabetic Retinopathy. Ophthalmology 114: 1884–1892 10.1016/j.ophtha.2007.02.023 17540447

[26] Haymore JG, Mejico LJ (2009) Retinal vascular occlusion syndromes. International ophthalmology clinics 49: 63–79 10.1097/IIO.0b013e3181a8db88 19584622

[27] Petzold A, Islam N, Hu HH, Plant GT (2013) Embolic and nonembolic transient monocular visual field loss: a clinicopathologic review. Survey of ophthalmology 58: 42–62 10.1016/j.survophthal.2012.02.002 23217587

[28] Maritim AC, Sanders RA, Watkins JB 3rd (2003) Diabetes, oxidative stress, and antioxidants: a review. Journal of biochemical and molecular toxicology 17: 24–38 10.1002/jbt.10058 12616644

[29] Tessari P, Cecchet D, Cosma A, Vettore M, Coracina A, Millioni R, et al (2010) Nitric oxide synthesis is reduced in subjects with type 2 diabetes and nephropathy. Diabetes 59: 2152–2159 10.2337/db09-1772 20484137 PMC2927936

[30] Sell DR, Monnier VM (2012) Molecular basis of arterial stiffening: role of glycation—a mini-review. Gerontology 58: 227–237 10.1159/000334668 22222677

[31] Nigro J, Osman N, Dart AM, Little PJ (2006) Insulin resistance and atherosclerosis. Endocrine reviews 27: 242–259 10.1210/er.2005-0007 16492903

[32] Barazzoni R, Zanetti M, Gortan Cappellari G, Semolic A, Boschelle M, Codarin E, et al (2012) Fatty acids acutely enhance insulin-induced oxidative stress and cause insulin resistance by increasing mitochondrial reactive oxygen species (ROS) generation and nuclear factor-kappaB inhibitor (IkappaB)-nuclear factor-kappaB (NFkappaB) activation in rat muscle, in the absence of mitochondrial dysfunction. Diabetologia 55: 773–782 10.1007/s00125-011-2396-x 22159911

[33] Cersosimo E, DeFronzo RA (2006) Insulin resistance and endothelial dysfunction: the road map to cardiovascular diseases. Diabetes/metabolism research and reviews 22: 423–436 10.1002/dmrr.634 16506274

[34] Steinberg HO, Chaker H, Leaming R, Johnson A, Brechtel G, Baron AD (1996) Obesity/insulin resistance is associated with endothelial dysfunction. Implications for the syndrome of insulin resistance. The Journal of clinical investigation 97: 2601–2610 10.1172/JCI118709 8647954 PMC507347

[35] Pfeifle B, Ditschuneit H (1981) Effect of insulin on growth of cultured human arterial smooth muscle cells. Diabetologia 20: 155–158 7009293 10.1007/BF00262020

[36] Davidson JA, Ciulla TA, McGill JB, Kles KA, Anderson PW (2007) How the diabetic eye loses vision. Endocrine 32: 107–116 10.1007/s12020-007-0040-9 17992608

[37] Wong TY, Klein R, Sharrett AR, Schmidt MI, Pankow JS, Couper DJ, et al (2002) Retinal arteriolar narrowing and risk of diabetes mellitus in middle-aged persons. Jama 287: 2528–2533 12020333 10.1001/jama.287.19.2528

[38] Carlson EC (1994) Scanning and transmission electron microscopic studies of normal and diabetic acellular glomerular and retinal microvessel basement membranes. Microscopy research and technique 28: 165–177 10.1002/jemt.1070280302 8068980

[39] Witt N, Wong TY, Hughes AD, Chaturvedi N, Klein BE, Evans R, et al (2006) Abnormalities of retinal microvascular structure and risk of mortality from ischemic heart disease and stroke. Hypertension 47: 975–981 10.1161/01.HYP.0000216717.72048.6c 16585415

[40] Klein BE, Klein R, McBride PE, Cruickshanks KJ, Palta M, Knudtson MD, et al (2004) Cardiovascular disease, mortality, and retinal microvascular characteristics in type 1 diabetes: Wisconsin epidemiologic study of diabetic retinopathy. Archives of internal medicine 164: 1917–1924 10.1001/archinte.164.17.1917 15451768

[41] Yang JY, Yang X, Li Y, Xu J, Zhou Y, Wang AX, et al (2016) Carotid Atherosclerosis, Cerebrospinal Fluid Pressure, and Retinal Vessel Diameters: The Asymptomatic Polyvascular Abnormalities in Community Study. PloS one 11: e0166993 10.1371/journal.pone.0166993 27907041 PMC5132305

[42] Chang YS, Weng SF, Chang C, Wang JJ, Tseng SH, Ko SY, et al (2016) Risk of Retinal Artery Occlusion in Patients With End-Stage Renal Disease: A Retrospective Large-Scale Cohort Study. Medicine 95: e3281 10.1097/MD.0000000000003281 27057891 PMC4998807

[43] Liew G, Wang JJ (2007) Retinal vascular signs in diabetes and hypertension—review. Arquivos brasileiros de endocrinologia e metabologia 51: 352–362 17505645 10.1590/s0004-27302007000200027

[44] Wong TY, Mitchell P (2007) The eye in hypertension. Lancet 369: 425–435 10.1016/S0140-6736(07)60198-6 17276782

[45] Bhargava M, Ikram MK, Wong TY (2012) How does hypertension affect your eyes? Journal of human hypertension 26: 71–83 10.1038/jhh.2011.37 21509040

[46] Gilbert AL, Choi C, Lessell S (2015) Acute Management of Central Retinal Artery Occlusion. International ophthalmology clinics 55: 157–166 10.1097/IIO.0000000000000087 26322435

[47] Duxbury O, Bhogal P, Cloud G, Madigan J (2014) Successful treatment of central retinal artery thromboembolism with ocular massage and intravenous acetazolamide. BMJ case reports 2014 10.1136/bcr-2014-207943 PMC4265031 25480141

[48] Canan H, Ulas B, Altan-Yaycioglu R (2014) Hyperbaric oxygen therapy in combination with systemic treatment of sickle cell disease presenting as central retinal artery occlusion: a case report. Journal of medical case reports 8: 370 10.1186/1752-1947-8-370 25399776 PMC4244145

[49] Fiess A, Cal O, Kehrein S, Halstenberg S, Frisch I, Steinhorst UH (2014) Anterior chamber paracentesis after central retinal artery occlusion: a tenable therapy? BMC ophthalmology 14: 28 10.1186/1471-2415-14-28 24612658 PMC3995909

[50] Schumacher M, Schmidt D, Jurklies B, Gall C, Wanke I, Schmoor C, et al (2010) Central retinal artery occlusion: local intra-arterial fibrinolysis versus conservative treatment, a multicenter randomized trial. Ophthalmology 117: 1367–1375 e1361 10.1016/j.ophtha.2010.03.061 20609991

